# Crystal Structure of CYP2B6 in Complex with an Efavirenz Analog

**DOI:** 10.3390/ijms19041025

**Published:** 2018-03-29

**Authors:** Manish B. Shah, Qinghai Zhang, James R. Halpert

**Affiliations:** 1 School of Pharmacy, University of Connecticut, Storrs, CT 06269, USA; james.halpert@uconn.edu; 2 Department of Pharmaceutical Sciences, Albany College of Pharmacy and Health Sciences, 106 New Scotland Avenue, Albany, NY 12208, USA; 3 Department of Integrative Structural and Computational Biology, The Scripps Research Institute, 10550 North Torrey Pines Road, La Jolla, CA 92037, USA; qinghai@scripps.edu

**Keywords:** cytochrome P450, CYP2B6, efavirenz analog

## Abstract

The over two dozen CYP2B structures of human, rabbit, and woodrat enzymes solved in the last decade have significantly enhanced our understanding of the structure-function relationships of drug metabolizing enzymes. More recently, an important role has emerged for halogen-π interactions in the CYP2B6 active site in substrate selectivity, explaining in part the preference for halogenated ligands as substrates. The mechanism by which such ligands interact with CYP2B enzymes involves conserved phenylalanine side chains, in particular F108, F115, or F297, in the active site, which form π bonds with halogens. To illustrate such halogen-π interactions using drugs that are major substrates of CYP2B6, we present here a crystal structure of CYP2B6 in complex with an analog of the widely used anti-HIV drug efavirenz, which contains a methyl group in place of the carbonyl oxygen. The chlorine of the efavirenz analog forms a π bond with the aromatic ring of F108, whereas the putative metabolism site on the distal end of the molecule is oriented towards the heme iron. The crystal structure showcases how CYP2B6 accommodates this important drug analog of considerable size in the active site by movement of various side chains without substantially increasing the active site volume. Furthermore, the CYP2B6-efavirenz analog complex provides a useful platform to investigate computationally as well as biophysically the effect of genetic polymorphisms on binding of the widely studied efavirenz.

## 1. Introduction

The cytochrome P450 (CYP) enzymes found on the membrane of the endoplasmic reticulum are a superfamily of heme-containing monooxygenases involved in the biotransformation of drugs and other xenobiotics [1]. Out of the 57 human CYP enzymes, about a dozen are involved in drug metabolism, including enzymes from the CYP1, 2 and 3 families [2]. Over the past several decades, our laboratory has focused on characterizing the CYP2B subfamily of enzymes in the absence and presence of a wide array of xenobiotics using functional, structural, computational, and biophysical methods [3,4,5,6,7]. The functional differences observed among these enzymes, which include rat CYP2B1, rabbit CYP2B4, human CYP2B6, dog CYP2B11, and woodrat CYP2B35 and 37, have allowed in-depth exploration of structure-function relationships of direct relevance to drug metabolism by other CYP families [8]. CYP2B6 shares at least 75% amino acid sequence identity with other members of the subfamily, constitutes up to 10% of the functional CYP enzymes in human liver, and is responsible for the metabolism of 10–12% of all drugs currently available on the market [9]. Moreover, CYP2B6 is a highly polymorphic enzyme, and variants of this enzyme have shown altered metabolism of many clinical drugs including cyclophosphamide, propofol, nevirapine, and efavirenz [10]. CYP2B6 also plays an important role in the bioactivation of many procarcinogens and environmental toxicants such as polychlorinated biphenyls, chlorpyrifos, and polybrominated diphenyl ethers [11,12,13].

Efavirenz is a non-nucleoside reverse transcriptase inhibitor commonly prescribed in the treatment of HIV [14]. CYP2B6 is the major catalyst of efavirenz oxidation and conversion to the most abundant metabolite 8-hydroxyefavirenz, which is then glucuronidated [15,16]. Furthermore, efavirenz is known to cause mechanism-based inactivation of CYP2B6 and can affect the expression of the enzyme [17,18]. A recent study investigated the chemical features of efavirenz that make crucial contacts in the active site of this enzyme [19]. The results further demonstrated the importance of the oxazinone ring and the ability of the CYP2B family of enzymes to metabolize certain efavirenz analogs predominantly compared to any other families of CYPs [20]. In this study, we report a crystal structure of CYP2B6 in complex with an analog of efavirenz. Because of the relatively low-affinity binding of efavirenz itself, the 2-desoxo-2-methyl analog that demonstrated high-affinity binding (*K_S_* = 0.21 ± 0.06 μM) was used for the structural studies reported here. The CYP2B6 used for expression, purification, structure determination and any other work in this study includes engineered mutations Y226H and K262R in the N-terminally truncated and modified construct with a His-tag at the C-terminus. The efavirenz analog (efavirenz 2-desoxo-2-methyl) is abbreviated as *I* throughout the manuscript for convenience purposes.

## 2. Results and Discussion

**Figure 1:**
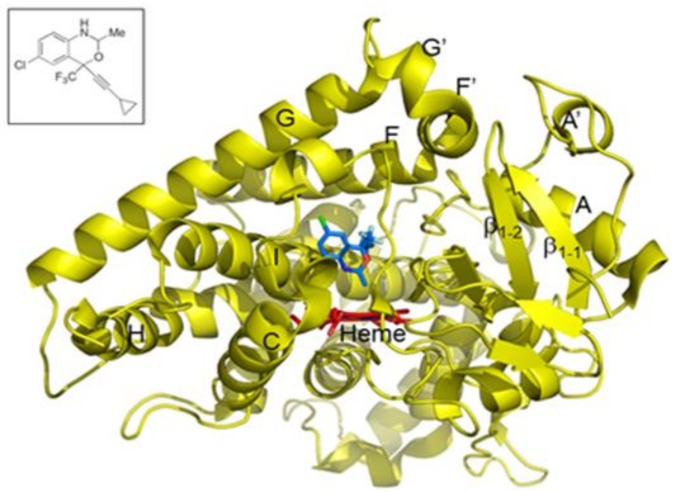
Crystal structure of CYP2B6 in complex with an efavirenz analog 2-desoxo-2-methyl (or *I)*. The structure of the compound, which substitutes a methyl group for the carbonyl oxygen in efavirenz, is shown in blue sticks in the active site near heme (red sticks). The inset represents the chemical structure of the efavirenz analog.

In our recent work, we demonstrated the role of halogen-π interactions in CYP2B6 ligand orientation through the use of halogenated and non-halogenated monoterpenes [21]. The presence of halogens clearly oriented the ligand predominantly toward certain phenylalanine side chains in the active site. To extend this study to halogenated drugs that are major substrates of CYP2B6, we determined the crystal structure of CYP2B6 in complex with an analog of the important anti-retroviral drug efavirenz. The 2-desoxo-2-methyl analog contains a methyl group in place of the carbonyl oxygen in efavirenz [20]. The crystal structure revealed how CYP2B6 accommodates this important drug analog in the active site near the heme (Figure 1). Interestingly, the chlorine of the efavirenz analog 2-desoxo-2-methyl (abbreviated as *I* for convenience) forms a π bond with F108 in a similar fashion to that illustrated in our recent study using monoterpenes [21]. The orientation of the methyl group towards the heme-iron corroborates the putative metabolic transformation at the oxazinone ring shown more recently by Cox and Bumpus [20]. The trifluoro group was located near the I101 and F115 side chains, whereas the cyclopropyl group was placed between the side chains of F206 and T302.

**Figure 2:**
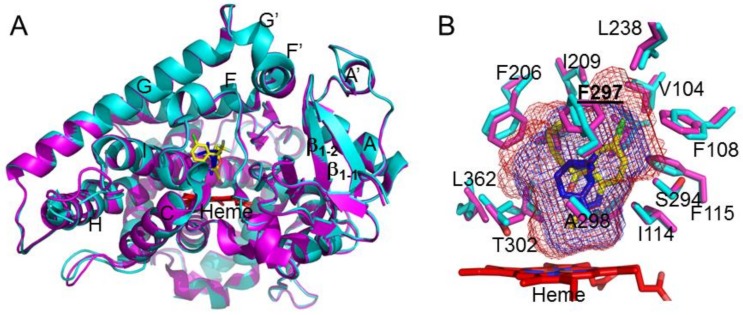
Structural overlay of CYP2B6-*I* (cyan) with CYP2B6-α-pinene (magenta) complex. (**A**) The closed conformation of the two CYP2B6 complexes superimposes onto each other with a root-mean square deviation of ~0.37 Å^2^. (**B**) Active site residue side chains located within 5 Å of the efavirenz and the α-pinene in the respective complexes are shown in stick representation. The F297 residue side chain that demonstrated altered orientation of the aromatic ring is labelled in bold with an underline. The cavity volume of CYP2B6-*I* (red mesh, 409 Å^3^) and the α-pinene (blue mesh, 311 Å^3^) calculated using Voidoo, represents the difference in the volume to accommodate the larger ligand in the active site.

Structural analysis of the CYP2B6-*I* complex and overlay with the previously solved amlodipine and α-pinene complexes revealed the differences and similarities in the overall conformation of the protein [3,22]. The CYP2B6-*I* structure adopted a closed conformation that is similar to the α-pinene complex (Figure 2A). The active site residue side chains superimpose onto each other, with the exception of the F297 side chain. The phenyl ring of F297 rotates ~90° to make room for the chloro group of the analog. In addition, there is small movement of the side chains, in particular F206 and F108, to accommodate the larger ligand in the active site compared with α-pinene. The analysis of the active site cavity volume did not reveal a substantial increase (409 Å^3^) upon binding *I* compared with the α-pinene complex (311 Å^3^) (Figure 2B) [3]. This indicates that subtle differences in the active site architecture of CYP2B6 allow binding of the much larger *I*, which contains multiple functional groups, in contrast to the pure hydrocarbon α-pinene.

**Figure 3:**
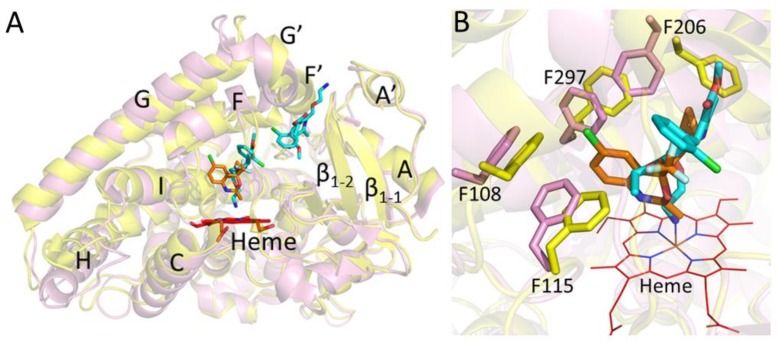
Structural overlay of CYP2B6-*I* (yellow) with CYP2B6-amlodipine (pink). (**A**) Differences in protein conformations upon binding efavirenz analog (orange, closed) compared to the amlodipine (cyan, open). (**B**) The active site shows significant reorientation of phenylalanines, in particular F206 and F297 to accommodate respective ligands. The Cl of the analog π bonds with F108 side chain.

Furthermore, the closed conformation of the CYP2B6-*I* structure was significantly different from the open conformation of the dual ligand complex with amlodipine (Figure 3A). The active site analysis displayed significant rotation of the side chains F206 and F297 compared with the amlodipine complex. As shown in Figure 3B, the F297 side chain is pushed away by more than 2 Å, in contrast to the amlodipine complex, and is likely to accommodate the chlorine group of *I* that is involved in the π-interactions with the F108 aromatic ring. Such movement of F297 perturbs the side chain of F206, which reorients by ~90° to assume an alternate position compared with that in the amlodipine complex [22]. Overall, the comparison of the CYP2B6-*I* complex and the previously solved structures of CYP2B6 with amlodipine and α-pinene clearly demonstrates that CYP2B6 can accommodate ligands of different size and shape by movement and reorientation of important phenylalanine side chains in the active site.

**Figure 4:**
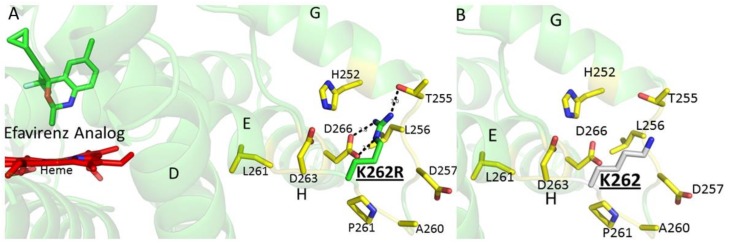
The residue side chain of arginine at position 262 in CYP2B6 makes hydrogen-bonding contacts with the side chains of T255 and D266 on the G and H helices, respectively.

It is important to note that all the crystal structures of CYP2B6 solved to date include an engineered mutation at amino acid residues Y226H and K262R for protein stability and solubility purposes [23]. The K262R amino acid substitution is a known single-nucleotide polymorphism denoted as CYP2B6*4 [24]. In addition, this substitution is present in a number of other CYP2B6 variants that include *6, *13, *16, *19, *20, *26, *34, *36, *37 and *38 and show altered metabolism of many clinical drugs, including efavirenz [25,26,27,28,29]. Interestingly, the majority of these variants, except *6 and *16, include at least one more substitution, namely Q172H. As shown previously, and observed in the current structure, the R262 side chain may play a crucial role in hydrogen bonding with neighboring helices, which could impact the enzyme structure and function (Figure 4) [23]. The K262 side chain in the wild-type enzyme may not be able to form similar hydrogen bonds with the neighboring residues. To conclude, the results provide important insights into the role of halogen-π interactions with active site aromatic side chains, which may aid in the orientation of drugs near heme conducive to metabolism by CYP2B enzymes. The CYP2B6-*I* complex also provides a structural basis to investigate, computationally and biophysically, the effect of genetic polymorphisms on binding of the widely studied anti-HIV agent efavirenz. In this context, it is worthy of note that the docking of the efavirenz analog into a model based on the crystal structure is consistent with both the major and minor metabolites of the compound.

## 3. Materials and Methods

### 3.1. Materials

Isopropyl β-d-1-thiogalactopyranoside (IPTG) and 5-cyclohexyl-1-pentyl-β-d-maltoside (CYMAL-5) were purchased from Anatrace (Maumee, OH, USA). 3-[(3-Cholamidopropyl) dimethylammonia]-1-propanesulfonate (CHAPS) was purchased from Calbiochem (EMD Chemicals, San Diego, CA, USA). Nickel-nitrilotriacetic acid (Ni2^+^-NTA) affinity resin and Macro-Prep CM cation exchange resin were obtained from Thermo Scientific (Rockford, IL, USA) and Bio-Rad Laboratories (Hercules, CA, USA), respectively. The pGro7 plasmid containing the GroEL/ES chaperones was obtained from Takara Bio (Shiba, Japan), and Amicon ultrafiltration devices were purchased from Millipore (Billerica, MA, USA). 3*α*,7*α*,12*α*-Tris[(*β*-d-maltopyranosyl)ethyloxy]cholane (232-chol) is a facial amphiphile synthesized according to a previous method [30]. Ampicillin, arabinose, δ-aminolevulinic acid (ALA), chloramphenicol, deoxyribonuclease I (DNase I), dithiothreitol (DTT), lysozyme, phenylmethylsulfonyl fluoride (PMSF), ribonuclease A (RNase A), 2-mercaptoethanol (β-ME), potassium phosphate, sucrose, tryptone, and yeast extract were purchased from Sigma-Aldrich (St. Louis, MO, USA). Glycerol, ethylenediaminetetraacetic acid (EDTA), and sodium chloride (NaCl) were obtained from Fisher Scientific (Waltham, MA, USA), and l-histidine was received from Spectrum chemicals. The *Escherichia coli* JM109 cells were from Agilent (Santa Clara, CA, USA). The efavirenz analog (efavirenz 2-desoxo-2-methyl) was purchased from Toronto Research Chemicals (North York, ON, Canada). All figures representing protein structures were created using PyMol (The PyMOL Molecular Graphics System, Version 1.5.0.4; Schrödinger, LLC, New York, NY, USA). The chemical structure was made using ChemDraw Ultra 10.0 (PerkinElmer, Chicago, IL, USA).

### 3.2. Protein Expression and Purification

The pKK plasmid containing the cDNA for human CYP2B6dH (Y226H/K262R) (*N*-terminally truncated and *C*-terminal his-tag with internal mutations at positions 226 and 262) and the pGro7 plasmid containing the GroEL/ES chaperones were co-transformed in *Escherichia coli* JM109 competent cells. The recombinant expression was carried out followed by purification using the protocol described previously [31,32]. In brief, an overnight Luria-Bertani broth (50 mL) of the plasmid transformed *Escherichia coli* (JM109) was used as a starting culture to inoculate Terrific Broth (1 L) supplemented with 20 mg/mL arabinose, ampicillin (100 μg/mL), and chloramphenicol (25 μg/mL). The culture was induced using IPTG (1 mM) and ALA (0.5 mM) at *A*600~0.7 at 37 °C. The cells were grown for 72 h at 190 rpm (30 °C) prior to centrifuging at 4000× *g*. The pellet was re-suspended in buffer containing 20 mM potassium phosphate (pH 7.4 at 4 °C), 20% (*v*/*v*) glycerol, 10 mM β-ME, and 0.5 mM PMSF followed by treatment with lysozyme (0.3 mg/mL, stirring for 30 min), and centrifugation at 7500× *g* for 30 min. After decanting the supernatant, the pellet was re-suspended in buffer containing 500 mM potassium phosphate (pH 7.4 at 4 °C), 20% (*v*/*v*) glycerol, 10 mM β-ME, 0.5 mM PMSF, 10 μg/mL RNase A, and 10 μg/mL DNase I before sonicating for 3 × 45 s on ice. At this stage, the detergent CHAPS was added (0.8% *w*/*v*), and the lysate was stirred for 30 min (4 °C) and ultra-centrifuged for 1 h at 245,000× *g*. The supernatant was collected, and the concentration of the P450 was measured using a difference spectrum of the ferrous carbonyl complex of the heme protein [33,34].

The supernatant was mixed with an equilibrated Ni^2+^-NTA resin and packed on a column, which was further washed with buffer containing 100 mM potassium phosphate (pH 7.4 at 4 °C), 100 mM NaCl, 20% (*v*/*v*) glycerol, 0.5% CHAPS, 10 mM β-ME, 0.5 mM PMSF, and 5 mM histidine. The protein was eluted using 50 mM histidine, and fractions containing the highest quality of protein (*A417*/*A280* ratios > 1) were pooled. The concentration of the P450 was determined using the reduced CO difference spectra. The pooled protein was further diluted 10-fold in 5 mM potassium phosphate (pH 7.4 at 4 °C), 20% (*v*/*v*) glycerol, 0.5% (*w*/*v*) CHAPS, 0.2 mM DTT, 0.5 mM PMSF, and 1 mM EDTA. This was then applied to a Macro-Prep CM weak cation exchange column, which was washed using 5 mM potassium phosphate (pH 7.4 at 4 °C), 20 mM NaCl, 20% (*v*/*v*) glycerol, 1 mM EDTA, and 0.2 mM DTT. The protein was eluted with 500 mM NaCl, and the fractions with the highest *A*417/*A*280 ratios (>2) were pooled. The concentration of the P450 was measured using the reduced CO-difference spectra. Spectral titrations performed with the 2-desoxo-2-methyl analog as described previously [35,36] yielded a type I spin shift with peak at 390 nm and trough at 417 nm and a *K*_S_ value of 0.21 ± 0.06 μM (4 replicates).

### 3.3. Crystallization and Data Collection

The pooled fractions of CYP2B6 from the CM column were further diluted to 18 µM in 50 mM potassium phosphate (pH 7.4 at 4 °C), 500 mM NaCl, 500 mM sucrose, 0.2 mM DTT and 1 mM EDTA. The efavirenz analog was added to a final concentration of 300 µM, and the samples were concentrated to 280 µM by centrifugation using 50 kDa cutoff Amicon ultrafiltration devices. The protein was again diluted to 18 µM using the above buffer containing 300 µM of the ligand, and the process was repeated twice before concentrating CYP2B6 to 280 µM. The protein-ligand sample was then supplemented with 4.8 mM CYMAL-5 and 0.028% (*w*/*v*) 232-cholate and filtered via 0.22 µm ultrafree centrifugal filters. Using the sitting drop vapor diffusion method, the crystals of CYP2B6-efavirenz analog were obtained over a period of 4–6 days at 18 °C in 0.8 M sodium formate, 0.1 M Tris pH 7.5, 8% *w*/*v* PEG 20,000, 8% *v*/*v* PEG 550 MME. The crystals were transferred to the mother liquor containing 20% sucrose for cryoprotection and the crystallographic data was collected remotely at Stanford Synchrotron Radiation Light (SSRL) source using beam line 14-1. Data were collected using 1-degree oscillations (240 frames) and 10-s exposure at 100 K, and images were integrated using iMosflm [37] and scaled using SCALA [38].

### 3.4. Structure Determination and Refinement

The structure of the CYP2B6 complex was solved at 2.99 Å resolution, using the previously determined 2B6-α-pinene complex (PDB ID 4I91) as the template. The Phaser program from the CCP4 suite was used for molecular replacement [39]. The solution was found in space group P1211 with six molecules per asymmetric unit. Iterative refinement was performed in Refmac5 [40] followed by model building using COOT [41] with *2Fo-Fc* and *Fo-Fc* electron density maps contoured to 1-σ and 3-σ, respectively, until the R-factor of 0.22 and R-free of 0.26 were obtained. The efavirenz analog with (2*R*,4*S*) diastereomer was modeled in to the observed unbiased electron density near the active site heme prior to the final steps of refinement (Figure S1). Attempts to model the (2*S*,4*S*) configuration were unsuccessful. Furthermore, the electron density near the cyclopropyl ring of the analog demonstrated disorder due to the lower resolution. Chains C and F showed the optimal density of the analog, whereas the other chains demonstrated varying degrees of disorder in electron density and higher B-factors. The coordinates and structure factors were submitted to the Protein Data Bank. Crystallographic data collection and refinement statistics are presented in Table S1.

## Supplementary Materials

Supplementary materials can be found at http://www.mdpi.com/1422-0067/19/4/1025/s1.
