# Supplementary material for: Crystal Structure of CYP2B6 in Complex with an Efavirenz Analog
Source: Int J Mol Sci. 2018 Mar 29;19(4):1025. doi: 10.3390/ijms19041025 (PMC5979536; doi:10.3390/ijms19041025)
Supplement: Supplementary file 1 [file ijms-19-01025-s001.pdf]

Crystal Structure of CYP2B6 in Complex with an Efavirenz Analog

Supporting Information

Manish B. Shah[1,2*], Qinghai Zhang[3] and James R. Halpert[1]

[1]Department of Pharmaceutical Sciences, University of Connecticut, Storrs, Connecticut 06269, United States. [2]Department of Pharmaceutical Sciences, Albany College of Pharmacy and Health Sciences, 106 New Scotland Avenue, Albany, New York 12208, United States. [3]Department of Integrative Structural and Computational Biology, The Scripps Research Institute, 10550 North Torrey Pines Road, La Jolla, California 92037, United States

*Corresponding author.

Contents

1. Figure S1: An unbiased electron density map……………………………3

2. Table S1: Data collection and refinement statistics………………………4

Figure S1: The *Fo–Fc* electron density map (contoured to 3σ, green mesh) obtained before modeling the ligand in the CYP2B6-efavirenz analog. The protein is shown in orange ribbons, ligand in yellow sticks, and heme in red sticks.

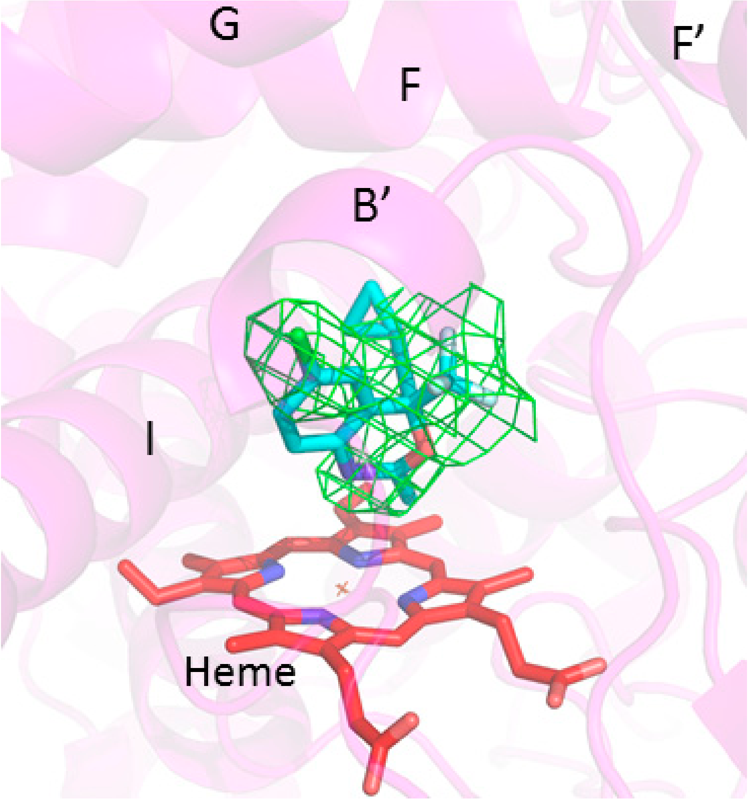

Table S1: Crystallographic data collection and refinement statistics.

Protein	CYP2B6dH
Ligand	Efavirenz Analog
PDB ID	5WBG
Crystal Space group	$P12_11$
**Crystal Unit Cell Parameters**	
a (Å)<br>b (Å)	103.29<br>197.78
c (Å)	119.22
$\alpha = \gamma$ (°)	90
β (°)	98.51
**Data Collection Statistics.** Values for highest resolution shell are shown in parentheses.	
Lightsource and beamline	SSRL 14-1
Wavelength (Å)	1.18
Resolution range (Å)	50.0-2.99<br>(3.06-2.99)
Completeness (%)	98.9 (96.7)
Redundancy	3.3 (3.2)
R_{merge}	0.18 (0.1)
Mean I/sigma (I)	6.4 (1.6)
Total reflections	89926
**Refinement Statistics.** Values for highest resolution shell are shown in parentheses.	
R-factor (%)	22 (33)
R_{free} (%)	26 (37)
**RMS Deviations**	
Bond lengths (Å)	0.01
Bond angles (°)	1.65
Average B factor ($Å^2$)	49.63
**Ramachandran Plot**	
Favored (%)	91.1
Outliers (%)	1.1
**Number of Atoms**	
Protein[a]	21849 (50.3)
Heme[a]	258 (41.3)
Solvent[a]	156 (39.5)
Efavirenz 2-desoxo, 2-methyl[a]	126 (97.1)
CYMAL-5[a]	181 (116.3)

[a]Average B-factors ($Å^2$) are in parentheses.
